# Supplementary material for: Applied phylogeography of Cyclopia intermedia (Fabaceae) highlights the need for ‘duty of care’ when cultivating honeybush
Source: PeerJ. 2020 Sep 2;8:e9818. doi: 10.7717/peerj.9818 (PMC7474521; doi:10.7717/peerj.9818)
Supplement: Supplemental Information 1 — Additional information regarding the spatial and ecological distribution of is provided. This includes photographs of the post fire refrigeration of C. intermedia and typical C. intermedia habitat. Image courtesy Gillian K. McGregor. [file peerj-08-9818-s001.pdf]

S1: *Cyclopia intermedia* distribution patterns with respect to the Cape Fold mountains.

The fynbos communities of the Cape Floristic Region are located either in high elevation mountainous sites (montane fynbos) or at low elevations in mountain valleys and sandy plains (lowland fynbos). *Cyclopia intermedia,* while having a relatively broad habitat tolerance, falls into the former category with a natural distribution limited to fynbos habitat in the mountains of eastern portion of the CFR (Fig S1). The Cape Fold mountains in the eastern CFR follow a latitudinal trend (Fig. S2) and support montane fynbos communities at higher elevations, while the eastern lowlands are often inhospitable to fynbos vegetation, which is replaced by arid shrub and woodlands. This is most apparent in the northern mountain ranges, however, coastal mountain communities are also isolated by deep river gorges dominated by woodlands (Fig. S3). This topographic complexity may thus be argued as a key isolating mechanism in the Cape, dissecting seemingly continuous populations into a mosaic of isolated stands.

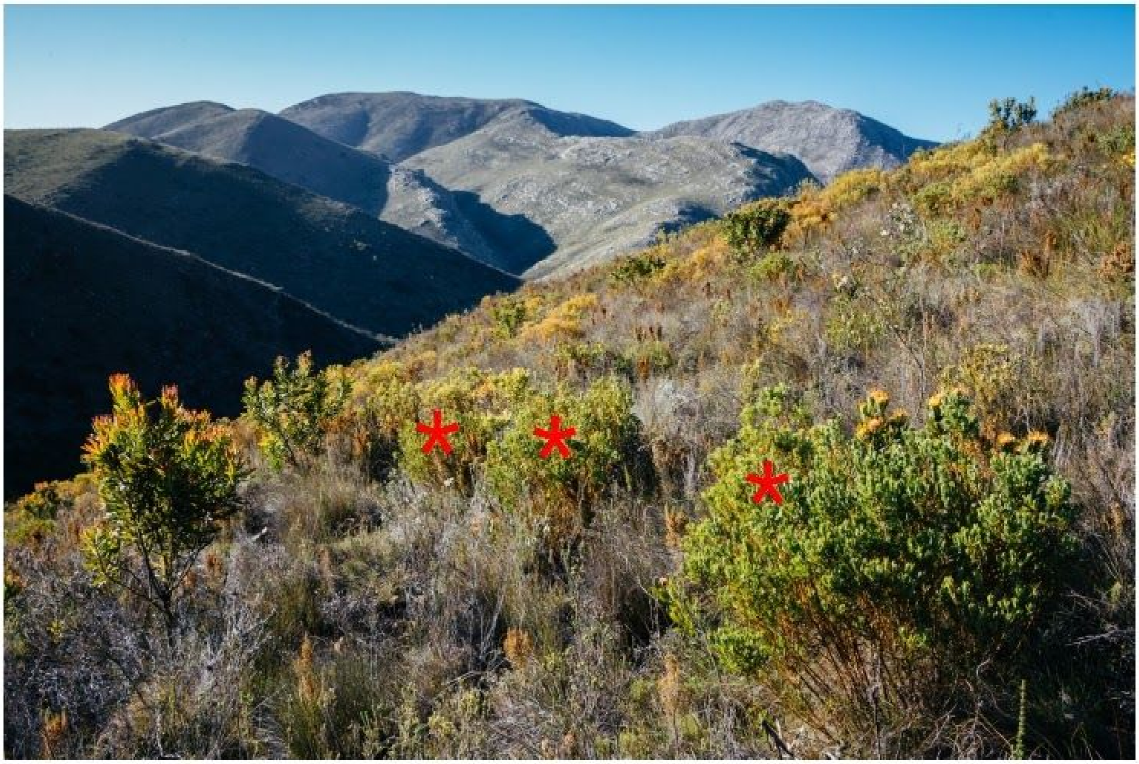

Fig. S1: Typical *Cyclopia intermedia* (indicated by *) montane fynbos habitat in the Langkloof (Kouga and Tsitsikamma mountains), Eastern Cape, South Africa. Image courtesy G. K. McGregor.

Fig. S2: High contrast Digital Elevation Map of the mountain ranges of the eastern CFR, including the sampling locations of the 17 *Cyclopia intermedia* populations included in the phylogeographic analysis.

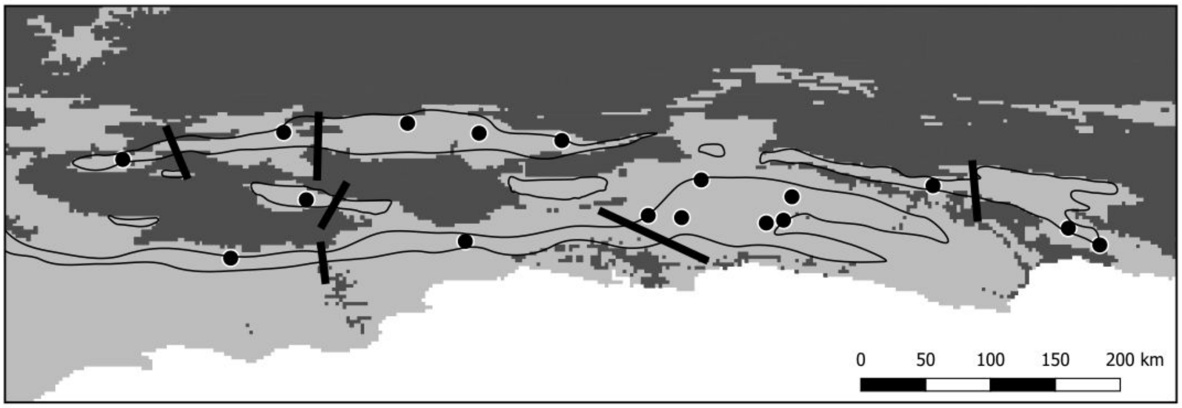

Fig S2: Vegetation map of the eastern CFR with the sampling location of the 17 *Cyclopia intermedia* populations included in the phylogeographic analysis provided. Light grey indicates fynbos habitat and dark grey is non-fynbos vegetation types. The montane fynbos habitat is outlined in black and deeply incised river gorgeous that dissect seemingly continuous montane fynbos are marked as thick black strips.

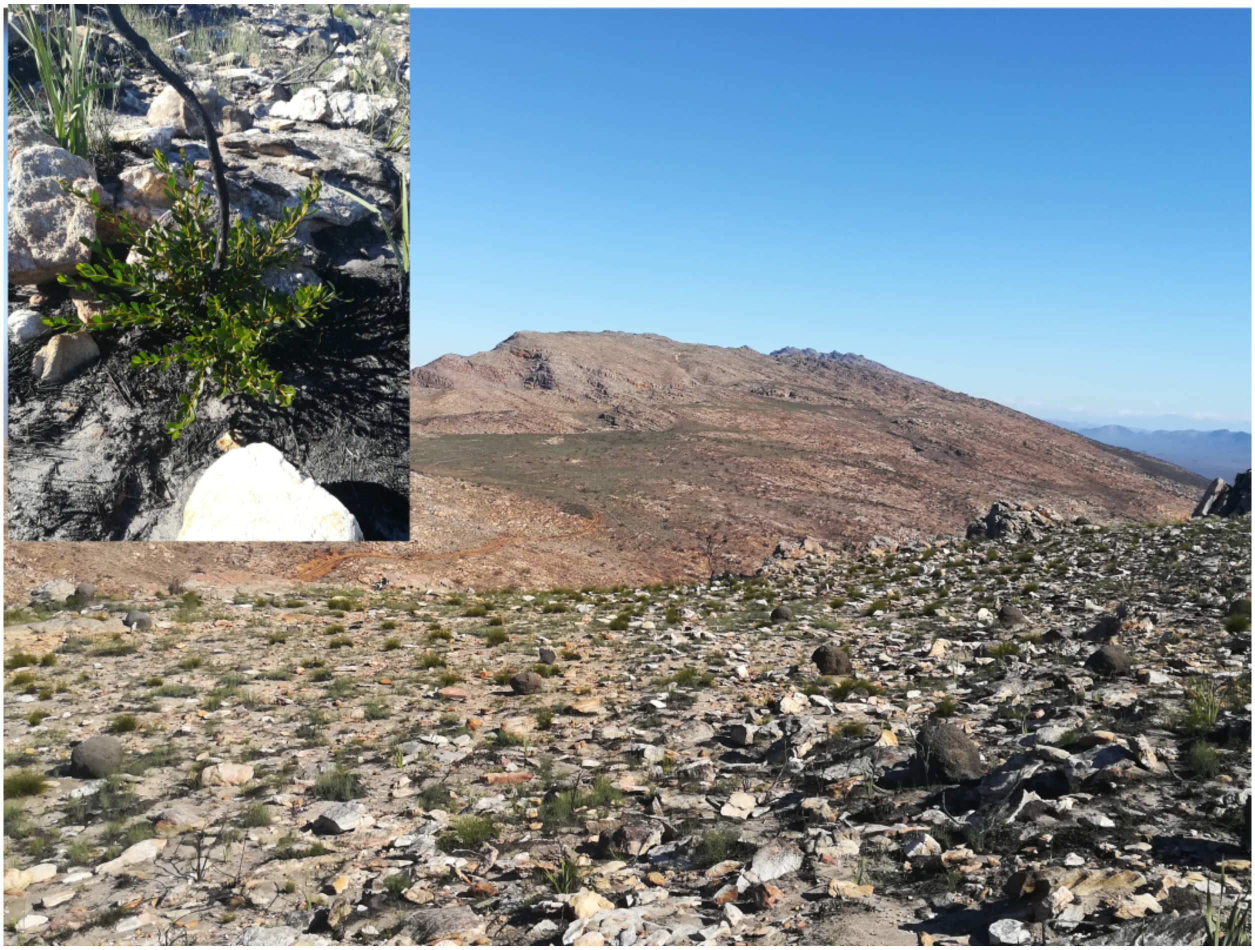

Fig. S4: *Cyclopia intermedia* habitat in the Anysberg post fire, a number of resprouting individuals (top left inset) were detected at the site using previously recorded GPS coordinates. Image: N. C. Galuszynski.
