# Supplementary material for: Applied phylogeography of Cyclopia intermedia (Fabaceae) highlights the need for ‘duty of care’ when cultivating honeybush
Source: PeerJ. 2020 Sep 2;8:e9818. doi: 10.7717/peerj.9818 (PMC7474521; doi:10.7717/peerj.9818)
Supplement: Supplemental Information 3 [file peerj-08-9818-s003.doc]

Table S1: PCR amplification protocol for the primers sources from Shaw et al. 2007.
